# From bits to bedside: entering the age of digital twins in cardiac electrophysiology

**DOI:** 10.1093/europace/euae295

**Published:** 2024-12-17

**Authors:** Pranav Bhagirath, Marina Strocchi, Martin J Bishop, Patrick M Boyle, Gernot Plank

**Affiliations:** Department of Cardiology, Amsterdam University Medical Center, Meibergdreef 9, Amsterdam, 1105 AZ, The Netherlands; School of Biomedical Engineering and Imaging Sciences, King’s College London, SE1 7EH London, UK; National Heart and Lung Institute, Imperial College London, London, UK; School of Biomedical Engineering and Imaging Sciences, King’s College London, SE1 7EH London, UK; Department of Bioengineering, University of Washington, Seattle, USA; Center for Cardiovascular Biology, University of Washington, Seattle, USA; Gottfried Schatz Research Center, Medical Physics and Biophysics, Medical University of Graz, Graz, Austria; BioTechMed-Graz, Graz, Austria

**Keywords:** Computational modelling, Digital twin, Personalized treatment, Electrophysiology, Atrial fibrillation, Ventricular tachycardia, CRT

## Abstract

This State of the Future Review describes and discusses the potential transformative power of digital twins in cardiac electrophysiology. In this ‘big picture’ approach, we explore the evolution of mechanistic modelling based digital twins, their current and immediate clinical applications, and envision a future where continuous updates, advanced calibration, and seamless data integration redefine clinical practice of cardiac electrophysiology. Our aim is to inspire researchers and clinicians to embrace the extraordinary possibilities that digital twins offer in the pursuit of precision medicine.

## Graphical Abstract

**Graphical Abstract:**
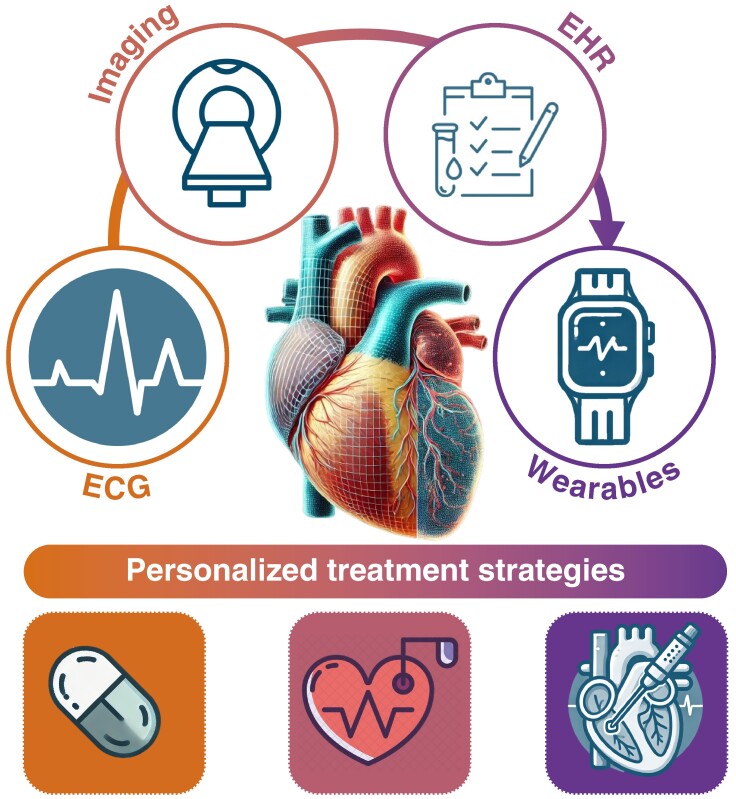
Personalized treatment strategies for arrhythmia care require the integration of various data sources and technologies such as structural information from MRI and CT, electrical information from ECGs, EHR data (e.g. medical history, test results, and medication use), and data from such as smartwatches containing real-time physiological parameters.

**What’s new?**

- A comprehensive discussion of the concept of physiological digital twins and their potential transformative power in the pursuit of precision medicine.
- An in-depth critique of the challenging calibration processes required to assimilate digital twins towards the goal of replicating patient behaviour.
- A review of current clinical applications of anatomical digital twins for use in risk stratification and pre-procedural planning for clinical therapies, including optimization pacing and resynchronization and treatment of atrial fibrillation or ventricular tachycardia.
- Identification and summary of further technological advancements and key improvement areas to overcome clinical implementation barriers.

## Introduction

Digital twins are virtual replicas of physical entities, systems, or processes, created using real-time data and advanced simulations, offering an innovative approach to monitor, analyse, and optimize performance across various industries, from manufacturing to healthcare. By bridging gaps between the physical and digital worlds, digital twins based on mechanistic modelling enable precise representation and prediction of behaviours, facilitating decision-making and enhancing efficiency.

In the realm of a rapidly digitizing healthcare sector,^1^ digital twins are emerging as a particularly transformative technology, including in cardiac electrophysiology. These advanced computational models simulate the electrical behaviour of the heart based on individual patient characteristics. Unlike conventional models, digital twins dynamically integrate anatomical, electrophysiological, and clinical data to create personalized representations of cardiac behaviour that evolve over time. By mimicking the dynamics of cardiac electrophysiological function in a virtual environment, digital twins have the potential to empower clinicians to explore underlying mechanisms of disease and tailor interventions of ever-increasing complexity to individual patient needs.

While current applications of personalized models have already demonstrated their potential in guiding treatment decisions and improving patient outcomes, the scope for further innovation and refinement is vast. This review aims to explore technological distinctions between different levels of personalization and/or twinning, including anatomical/structural and mechanistic aspects for personalized model adaptation. Subsequently, it investigates the calibration process required for digital twins. Challenges and costs associated with maintaining up-to-date digital twins are also discussed. In addition, current applications of personalized models in pacing and resynchronization therapy, atrial arrhythmias, and ventricular arrhythmias are discussed. This work proposes a bold future vision for mechanistic digital twins, identifying areas requiring improvement and technological advancements. We articulate a comprehensive research agenda for future developments, aiming to enhance the efficacy and applicability of digital twins in cardiac electrophysiology.

## What is a digital twin?

The origins of the modern concept of ‘digital twins’ can be traced back to work in the early 1990s by Prof. David Gelernter, who speculated that rapid development of software engineering and related fields would soon lead to the creation of ‘Mirror Worlds’,^2^ in which sophisticated computational systems could create convincing simulacra of real-world physical objects. Around a decade later, Prof. Michael Grieves articulated a refined version of this formalism, termed the ‘Mirrored Spaces Model’.^3^ This model envisions continuous bidirectional exchange of information between real and virtual spaces. A critical element of this framing is that applying a particular stimulus or perturbation should lead to the same emergent response, whether this is occurring in the real or virtual space. Since then, the use of digital twin terminology has become well established and pervasive, especially in the fields of manufacturing and industrial systems engineering. IBM’s information page on the topic (https://www.ibm.com/topics/what-is-a-digital-twin) provides a concise history of the field as well as a few key parameters used to semantically distinguish digital twins from computational models. In their framing, a digital twin ‘spans the object's lifecycle, is updated from real-time data and uses simulations, machine learning (ML), and reasoning to help make decisions’.

However, when it comes to the application of digital twin technology to biological systems, several challenges arise. Given the complex, dynamic, and unpredictable nature of biological systems, it is implausible that digital twins compliant with these strenuous definitions can be created using contemporary (or even future) clinical and computational approaches. Notions like creating virtual avatars that span human lifetimes or comprehensively reconcile multi-scale physiological inter-actions from atom to organism are simply not feasible. Likewise, many relevant aspects of physiology pose major barriers to functional twinning even over short timespans; to name a few: variability in heart rate due to sympathetic/vagal tone or circadian variability, fine grain tracking of drug effects, and pathophysiological changes during ischaemic events. Compared with the inanimate objects that inspired digital twin pioneers, living organisms are subject to countless fluctuations and vicissitudes arising from the choices they make—how they inter-act with the world (and vice-versa).

Nevertheless, as detailed in subsequent sections of this review, life science researchers have adapted key aspects of the digital twin formalism to be relevant in the context of human health. Each of these areas encompasses great opportunities for refinement, extension, and application as the field of digital twinning in healthcare grows. For instance, there has been robust interest in personalization of computational models via many vectors, including: anatomical structure; physiological inter-subject variability; disease-related remodelling; genetic background; attributes, placement, and programming of medical devices; and biomarker levels. This custom-tailoring of simulations based on individual features could plausibly be extended to account for consequences of changes in relevant biomolecule levels during a normal day (e.g. arising from changes in exercise, stress, diet, environment, etc.), varying levels of drug bioavailability due to dosing schedules, dynamic growth and remodelling of organ systems from physiological or environmental cues, ageing or epigenetic modification, etc. There is also an opportunity to create computational models with varying levels of complexity (ranging from simple pre-trained ML models to complex physics-driven simulations consisting of a set of governing equations) that actively ‘listen’ for updates on patient state entered into the electronic health record (e.g. new imaging data, time-dependent biomarker trends from blood draws or other diagnostic assays, major life events that alter physical or mental health, and data from wearables). These models could then automatically and rapidly ‘respond’ by pushing revised predictions, notifications, or prognoses that could be interpreted by the care team. Many of the building blocks needed to create simulation frameworks with these digital twin-like attributes already exist, so the field is poised for growth in the coming years.

To ensure this growth proceeds in a responsible and productive way, we believe caution must be exercised to avoid cavalier use of the digital twin vernacular by biomedical researchers. Given the robust literature and stringent conventions of the traditional (i.e. manufacturing and industrial systems) digital twin formalism outlined above, use of this terminology may be risky when applied to human physiology and could prove misleading to consumers of scientific information (especially members of the public), since it threatens to set unrealistic expectations. Re-branding personalized models as ‘digital twins’ when the virtual space inherently involves some simplification of the physical space undermines a central tenet of modern computational physiology: namely, that we can often learn as much about a complex system by considering the elements *deliberately left out of* a model as we can from components that *are* included. On the other hand, the push towards precision medicine enabled by model systems that more accurately recapitulate physiological reality is a welcome development. If the digital twin terminology is deployed responsibly with due attention paid to clearly outlining blind spots and limitations of each model, it is reasonable to expect the usefulness and trustworthiness of such models to steadily improve. This is especially salient given increasingly wide-spread collection of precise measurements of patient data, which stands to greatly improve our understanding of complex physiological phenomena against the backdrop of considerable inter- and intra-patient heterogeneities.

### Guidelines for specific digital twins descriptions

In this spirit, we propose two common-sense guidelines to be followed for digital twins in healthcare. First, when presenting aspects of model personalization, it is critical to explicitly outline which physiological attributes from the physical space are reproduced by tuning which parameters of governing equations in the virtual space; likewise, the most salient aspects of the physical space that are *not* personalized in the virtual space should be foregrounded. Second, researchers should take care to apply appropriate qualifiers when describing digital twins to reflect the degree to which the virtual and physical spaces are inter-twined. We suggest a three-level classification system for applications in the context of computational modelling of cardiac electrophysiology:

1. The ‘mechanistically related’ physiological model: e.g. simulations based on first principles that recapitulate relevant electrophysiological mechanisms at play, *qualitatively similar* to how such mechanisms unfold in real physical hearts; This class of models is loosely constrained with generously broad parameter spaces to endow them with the capability to produce all emergent phenomena inherently embedded in the model (produce virtual electrode polarizations under a given stimulus, induce an arrhythmia under a given protocol, terminate an arrhythmia for a given shock, etc). As the relation between model predictions and clinical data are qualitative, this class of models deviates from the formal definition of a digital twin as used in other fields. Nonetheless, at the current state of the art this appears to be the most prevalent type of computational model used in cardiac electrophysiology applications.
2. The ‘functionally similar’ digital twin: e.g. simulations that satisfy the above criteria while also being calibrated to be *representative of a patient cohort* rather than of an individual patient, therefore facilitating generic interpretation of observations in real physical hearts and generic prediction of responses to relevant therapeutic interventions. In this context, virtual cohorts can be defined as an ensemble of functionally similar digital twins representative of a specific population.^4^ These are subject to more rigorous constraints imposed by observable data that render them specific and representative for a narrower group of patients, characterized by an inferred variability in factors such as body composition, cardiac anatomy and structure, pathology, and electrophysiological behaviour (wavelength, restitution, etc). Such virtual cohorts are suitable to make ensemble predictions as relevant for the key quantities of interest (defibrillation threshold, pacing capture threshold, arrhythmia inducibility, and termination). Predictions of such models must then fall within the expected distribution of observations in the real patient cohort they aim to replicate. For trustworthy applications, regulatory evidence thereof must be provided. A rapidly growing area of applications of this type of models is in medical device and therapy development (e.g. to predict the efficacy of a novel device design^5^ or therapeutic protocol^6^ in each target patient population). Using a virtual cohort as a large assembly of digital twins in the form of *in silico* clinical trials^7^ offers unprecedented opportunities and promise to overcome limitations of traditional means for evaluating treatment efficacy and safety, which rely on pre-clinical testing in (non-human) animal studies and expensive and time-consuming clinical trials. Once sufficient evidence is generated demonstrating key features in functionally similar virtual cohorts, the resulting data could potentially be used in regulatory submission and facilitate device testing in *in silico* clinical trials where the number of patients enrolled, and the duration of a real clinical trial can be significantly reduced.
3. The ‘functionally equivalent’ digital twin: e.g. simulations conducted in models that match cardiac anatomy, structure, and are *quantitatively calibrated* to match functional observations from an individual patient’s heart in a one-to-one manner under a broad range of physiological circumstances and testable conditions, which can be employed to make rigorous and specific predictions for that patient’s treatment. By matching the exact cardiac anatomy and functional characteristics of an individual’s heart, this class of digital twins allows for precise and personalized treatment planning, ensuring that interventions are tailored to the unique physiological conditions of that patient. Functionally equivalent digital twins are required where it must be ascertained that real physical and simulated *in silico* behaviour are causally related, not merely correlated. If this condition is met, insights gained from *in silico* analysis of risk or therapy outcome can and should be used to inform clinical decision making.

This classification facilitates a clearer understanding of the degree of personalization and fidelity involved in each class of models. As healthcare researchers transition from understanding the foundational concepts of digital twins to exploring the practical challenges of their implementation, it is crucial to address the complexities involved in model calibration. The next section will explore how achieving accurate and reliable calibration is vital for the successful application of digital twins in healthcare.

## Model calibration and evolution

### Requirements and challenges of model calibration

Requirements on comprehensiveness and fidelity of model calibration vary according to the aspired classification of a digital twin and the intended application. Cardiac function emerges from an interplay between multiple factors including gross anatomy (shape and mass), tissue type and structure (viability, fibrosis, fat, and cardiac conduction system), and associated functional physical properties (excitability, refractoriness, conduction velocity, anisotropy, etc). In a model, these features are governed by parameters, which must be determined from observable data, either by direct measurement or by indirect inference. Whether model predictions are qualitatively and quantitatively correlated to the physiology observed in the real physical space under any specific circumstances critically depends on the care taken during the calibration procedure. As the parameter space of electrophysiological models of the heart is extremely high-dimensional, unsuitable, or insufficiently stringent calibration constraints would make any interrogation of a digital twin unreliable, permitting the model to predict any (physiological or unphysiological) outcome. That is, while one choice of parameters may predict response to pacing, inducibility, maintenance of an arrhythmia, or defibrillation, another—potentially similar—choice may predict the exact opposite. As such, accurate model calibration is key, but technically challenging to achieve as most model parameters elude direct measurement *in vivo*. Even *ex vivo* measurements are often unfeasible or afflicted with vast uncertainties.^8^ Moreover, the relation between parameters and model outputs is non-unique. Not all parameters may be identifiable, and the sheer size of the parameter space renders model calibration computationally expensive.

### Technological constraints in cardiac electrophysiology models

An additional and often underappreciated complexity is represented by the technological constraints imposed by biophysical models of cardiac electrophysiology, based on first principles reaction-diffusion type formulations such as the cardiac mono- or bi-domain model.^9^ The fast upstroke of an action potential translates into a steep depolarization wave front in space, imposing constraints on the spatio-temporal discretization. In general, to obtain a sound representation, discretization of <500 µm and <50 µs in space and time are required, respectively, rendering the evaluation of such models computationally expensive.^10^ This is particularly problematic in tissue substrate of compromised electrophysiological function such as in an infarct border zone where conduction velocity is reduced. In these areas, the spatial extent of depolarization wave fronts shrinks to sub-cellular dimensions of <100 µm, which requires adequate spatial resolution. Relaxing these constraints causes numerical artefacts such as ultra-slow conduction or artificial conduction block that render the computational model unreliable. Models may then predict behaviours such as non-sustainability of an arrhythmia or microscopic functional re-entrant arrhythmia that do not correspond to any physiological correlate in a modelled patient. Owing to the inherent complexity of organ scale electrophysiological models, such artefacts often go unnoticed and are interpreted, erroneously, as physiological findings. These aspects are currently under-investigated and poorly understood, but alerting concerns regarding the validity of predictions in modelling studies where model accuracy may have been traded in for computational efficiency are emerging. As a point in case, it has been demonstrated that the outcome of arrhythmia induction and sustainability in inadequately discretized models is essentially random.^11,12^

These problems are mitigated by using adequately high spatio-temporal resolutions at the cost of increasing the computational costs. For instance, increasing spatial resolution by a factor of two results in an almost 10-fold increase in compute costs. Even with a coarse discretization of 400 µm as used in recent clinical applications in VT risk stratification,^13^ VT ablation,^14^ or AF ablation,^15^ compute expenses incurred are on the order of days, even when using advanced high performance computing resources. These factors that pose a significant roadblock towards clinical application of cardiac electrophysiology models triggered the development of alternative more lightweight model formulations, such as the hybrid reaction-eikonal model, where discretization constraints are lifted.^16^ Such models execute in real-time and can be used, e.g. to simulate VT and associated ECGs or electrograms (EGMs) that can be related to clinical observations.^17,18^

### Digital twin snapshots—calibration for an instant in time

Real digital twins require updates over time, either continuously or periodically, where each update taken at a given instant in time refers to a digital twin snapshot. For a first snapshot, available data may be more abundant as this may happen during a comprehensive clinical assessment, whereas later snapshots might be more lightweight using less data, e.g. ECGs from wearables, device EGMs. Thus, the creation of true digital twins relies on the repetitive acquisition of snapshots.

**Figure 1:**
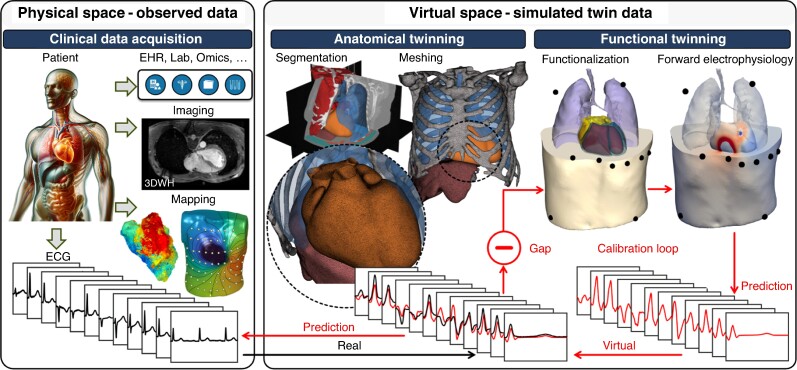
Calibration of a digital twin. Generalized workflow for the generation of a digital twin of cardiac electrophysiology. Clinical data acquired from a given patient including imaging (MRI, CT), sensor data (ECG, electro-anatomical mapping, body surface potential maps) and health data are used to build a virtual patient. Twinning workflows typically comprise two stages: (1) The anatomical twinning stage involves multi-label segmentation of clinical imaging data to generate computational meshes representing relevant anatomy, typically the heart or parts thereof, embedded in a torso. (2) The functional twinning stage involves functionalization, i.e. providing a reference frame for defining the space-varying parameter fields to be identified, the evaluation of a physiological forward model to compute predictions of the observed clinical data such as the ECG, and the measurement of the discrepancy between prediction and real physical observation. Parameter fields are then adjusted iteratively to reduce the gap between observations in the virtual and the physical space until the gap is deemed small enough such that the digital twin can be considered to behave sufficiently similar to the patient. For a functionally equivalent digital twin this calibration step must be followed by a validation step where the causal correspondence between digital twin and patient must be demonstrated by showing that the observed response to a perturbation such as, for instance, pacing is sufficiently similar.

In general, procedures for generating snapshots of physiological digital twins are separated into two distinct stages, an anatomical and a functional twinning stage (*Figure 1*). Anatomical twinning refers to procedures for turning tomographic image data into a structurally accurate representation of a patient’s heart (or parts thereof, for example single (or bi-) atria or ventricle, full 4-chamber models). An important step is image segmentation, in which clinical images are analysed to identify voxels comprising different cardiac regions and other organs of interest, and to sub-differentiate types of tissue (viable, fibrosis, and fat) within segmented classes. Current modelling studies use semi-automatic techniques that require manual processing, introducing operator bias and yielding simplified anatomical models,^19,20^ with a trend towards more advanced automated segmentation based on ML, which may yield anatomically more accurate models.^21^ The challenge of converting image-based segmentations into discrete computable meshes has been addressed^22^ and streamlined^23,24^ to the extent that the resulting models are compatible with clinical applications. Challenges in identifying anatomical and structural features from 3D images persist, but improved image acquisition modalities^25^ and advanced ML based segmentation techniques^21^ will eventually yield nearly 1:1 reproductions of patient anatomies.^21,26^

### Functional behaviour and structural remodelling

Many key aspects of the functional behaviour of digital twins heavily rely on faithful representation of structural remodelling (specifically scar, fibrosis), particularly important in the simulation of arrhythmia dynamics. The current gold-standard imaging modality for tissue-type identification for reconstruction into computational models remains late gadolinium enhanced (LGE)-MRI, capable of delineating dense scar along with adjacent border-zone tissue.^27^ In chronic ischaemic cardiomyopathy, cardiac CT is increasingly being used to accurately quantify myocardial wall thinning, which is closely associated with dense transmural scarring in this pathology,^28,29^ providing an opportunity to embellish CT-derived models with fibrotic remodelling. Advances in fully 3D LGE-MRI imaging and development of wideband sequences to mitigate implanted device artefacts are set to enhance accurate digital twinning of structural remodelling in the coming years.^30,31^ Research in late-enhancement MRI contrast agents,^32^ advances in T1-mapping capabilities,^33^ and analysis of extracellular volume imaging (from both CT and MRI^34,35^) are also set to improve our ability to incorporate different types of fibrosis and textures into models, which are known to have their own intricate electrophysiological properties. Moreover, new approaches are emerging that will enable imaging of other potentially relevant drivers of pro-arrhythmic substrate (e.g. Dixon MRI sequence for epicardial adipose tissue^36^ or PET techniques for characterizing inflammation and/or sympathetic innervation).^37^ In CT, the development of photon-counting CT scanners has the real potential to overtake all other imaging modalities as the method of choice for digital twin creation. Paracellular resolution along with highly specific tissue typing may render other limitations obsolete (except for issues related to implanted device-related imaging artefacts and radiation dose to the patient), possibly allowing all the anatomical detail of a given scar substrate to be imaged and reconstructed into a patient-specific model with minimal effort.

### Overcoming functional twinning challenges

The fundamental challenge in functional parameterization of cardiac digital twins is to infer a high dimensional parameter vector governing the electrophysiological behaviours of a complex computational model from sparse, noisy and uncertain observations obtained non-invasively (e.g. ECGs or body surface potential maps) or invasively (e.g. discrete EGMs or electro-anatomical maps),^38^ such that differences between simulated and physical reality are minimized. At the same time, important technical constraints must be honoured to ensure sound and valid model predictions. This is an ill-posed inverse parameter identification problem constituting the fundamental core challenge of *functional twinning.*

Specifically, observations of emergent electrophysiological properties (conduction velocities, action potential duration, and restitution behaviour) are limited in accuracy and are thus ill-suited for constraining model parameters. However, numerous model parameters must be identified from these or assumed. Key parameters to identify include fibre architecture^39–41^; orthotropic conductive tissue properties governing anisotropic current flow and the speed of wavefront propagation; spatial heterogeneity in excitability and intrinsic properties of cellular dynamics such as action potential and restitution properties; and the makeup of the cardiac conduction system.^42,43^ None of these parameters can be wholly characterized with contemporary tools. Therefore, their distribution in the human population or in specific patient cohorts is not well characterized. An additional level of complexity arises from the heterogeneous parameterization of anatomical models based on the tissue type identified from imaging. Specifically, tissue-type alone does not provide adequate information on its functional viability or corresponding (dynamic) functional properties, which play important roles in the pathological responses these models aim to replicate.

Most current computational electrophysiology studies claiming to use cardiac digital twins refer to anatomical, and potentially, structural digital twins, but refrain from any functional calibration.^44^ Rather, parameters are assumed based on literature values and kept constant, thus ignoring any inter-subject functional variability (let alone intra-subject heterogeneity). Personalization based on existing ECG or body-surface potential mapping may provide useful initial estimates of functional properties in sinus rhythm but may not be adequate for the simulation in the context of arrhythmia, where spatial heterogeneity and restitution of properties such as conduction and repolarization are key in governing dynamics. Direct like-for-like comparison to observable clinical phenotypes are seldom shown, as these models predict clinical signals that reveal a significant gap between the virtual and physical space.

While challenging, major efforts are under way to solve the functional twinning problem. Key technologies include: ultra-fast forward electrophysiology models predicting electrical sources and potential fields with real-time performance^16,45–47^ sensitivity studies performed to identify which parameters have the most consequential effects on model predictions, in order to pinpoint the most relevant calibration data required^48^; physiological constraints integrated in the identification procedure to cope with non-uniqueness^49^; anatomical reference frames^50,51^ to facilitate the automation of parameter sweeps, combined with advanced optimization approaches.^52,53^ The latest studies have shown the feasibility of creating digital twins that replicate like-for-like a patient’s ECG,^45,54^ but the use of digital twins to predict patient responses under conditions not accounted for during model calibration remains uncharted territory.

### True digital twins—repetitive calibration with longitudinal data

Maintaining up-to-date digital twins constitutes an even more challenging problem compared with creating a digital twin snapshot. Constantly updating the model to reflect ongoing physiological changes, disease progression, and treatment effects requires continuous—or at least periodic—integration of new data and recalibration of parameters. Such continuously monitored data e.g. from wearables are sparse and less comprehensive than those acquired clinically (e.g. single lead ECGs), rendering parameter inference even more challenging. This dynamic aspect of digital twinning demands robust, adaptive algorithms capable of real-time data assimilation and model adjustment. Advances in computational power, ML, and data acquisition technologies (from wearables or implanted devices) are pivotal in overcoming these hurdles, enabling the creation of cardiac models that are predictive, personalized, and dynamically responsive.

## Current clinical applications

Digital twins hold immense promise in revolutionizing the field of cardiac electrophysiology, offering a paradigm shift towards precision medicine. In current clinical practice, their applications span various areas, each contributing to enhanced understanding of mechanisms behind patient response, leading to improved patient care and treatment outcomes. However, as described in the following sections, these are currently limited to *mechanistically related* and *functionally similar* digital twins, leaving the potential of a *functionally equivalent* digital twin unexplored.

**Figure 2:**
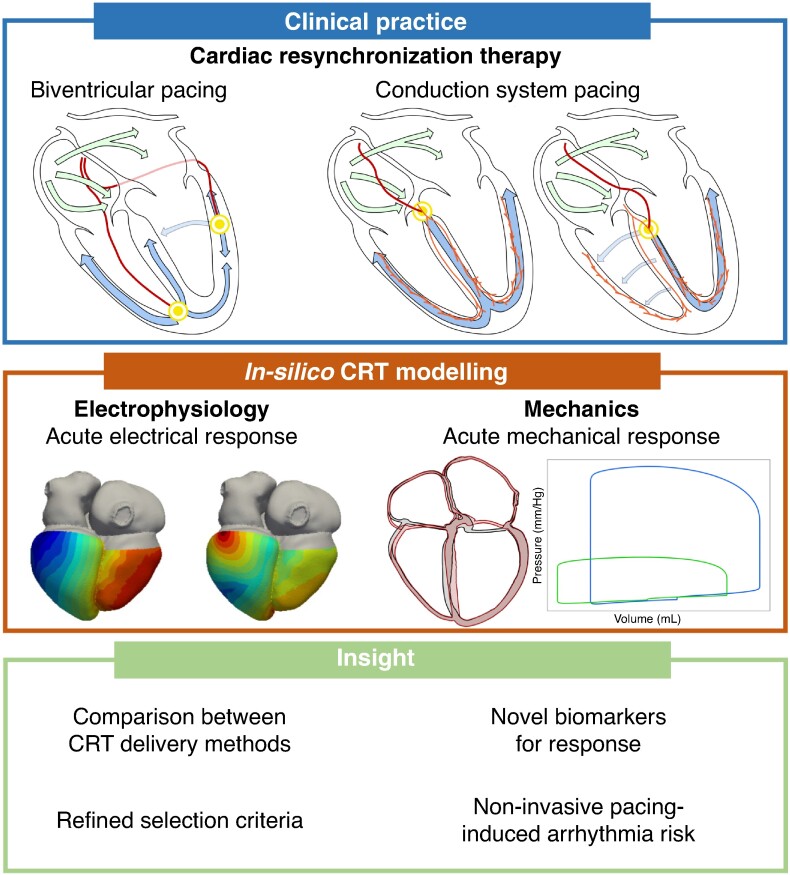
Clinical application of computational modelling for CRT. Top panel illustrates the different concepts and mechanisms of resynchronization therapy. Middle panel demonstrates computational modelling derived simulations of electrical activity and mechanical/hemodynamic response during resynchronization therapy. The lower panel presents the key insights that can be gained when applying computational modelling.

Pacing and resynchronization^55^ is used in patients with left ventricular (LV) dyssynchrony,^56^ caused by a range of conduction disturbances, from proximal or distal left bundle branch block to diffuse LV conduction slowing. In clinical practice, cardiac resynchronization therapy (CRT) is delivered through biventricular pacing (BVP) (*Figure 2*).

However, more recently, technical improvements have emerged to achieve CRT via His bundle pacing (HBP) or left bundle branch pacing (LBBP), which involve stimulating the heart’s specialized conduction system directly.^57^ Despite these innovations, 30–50% of patients receiving CRT do not respond to therapy.^58^ These clinical challenges have made CRT the subject of many computational studies.

Different types of computational models have been used to simulate CRT effects on cardiac function. Mechanistically similar digital twins allowed investigation of the response to BVP and refinement of patient selection criteria based on sex^59^ and identification of mechanisms for poor response to CRT.^60^ Insights on reduced response to BVP have also been enabled by functionally equivalent digital twins validated against patient data not used during model calibration, although only the simulated response to BVP was independently validated.^61^ Additionally, functionally similar digital twins have been used to identify predictive biomarkers of long-term response^62^ and conduct *in silico* trials to compare different pacing modalities (HBP vs. LBBP vs. BVP), highlighting the importance of substrate identification and patient selection to optimize outcomes.^63,64^ In terms of cardiac repolarization, computational studies have used functionally similar digital twins to explore changes induced by pacing modalities, such as BVP, on local repolarization times and the T-wave.^65–67^ These studies provide insights into the acute effects of pacing on repolarization dynamics and arrhythmogenic risk, particularly in patients with heart failure and ischaemic cardiomyopathy. However, there is a gap in research regarding the effects of HBP and LBBP compared with BVP on repolarization changes, highlighting an area for future investigation. Despite the contributions of computational modelling to our understanding of different aspects of CRT response, functionally equivalent digital twins have not yet been deployed in clinical settings. Full realization of this goal would require the development of real-time updatable digital twins that can track the patients’ hearts post-implantation and aid in personalized treatment decisions based on long-term rather than acute predictions. Such models could enable testing of various pacing modalities and facilitate precision medicine based on *in silico* predictions of long-term response.

### Atrial arrhythmias and risk of stroke

Atrial arrhythmias are characterized by irregular electrical activity, leading to ineffective contraction and potential blood stasis, pre-disposing individuals to thromboembolic events like stroke. Despite advancements in treatment modalities, the identification of patients with elevated stroke risk remains challenging.^55^

**Figure 3:**
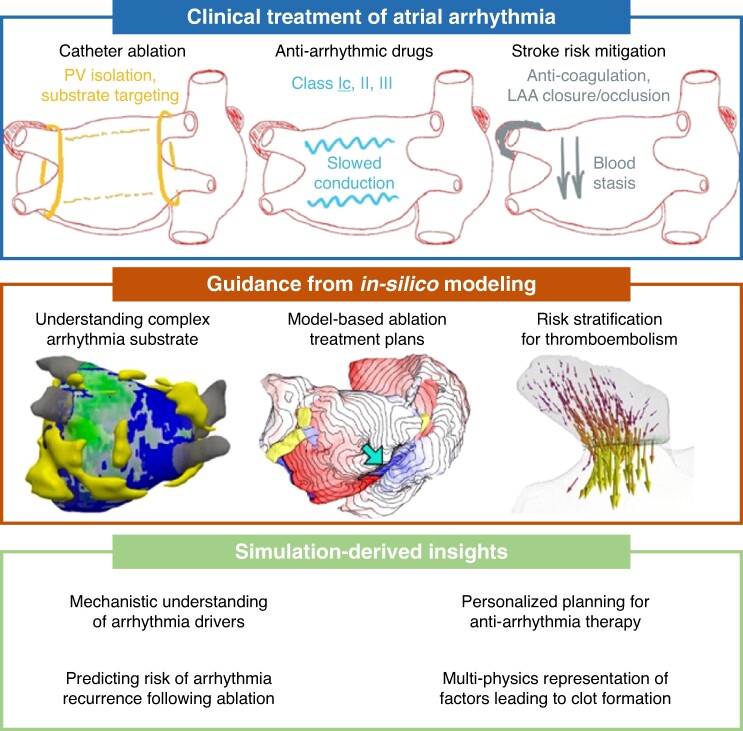
Clinical application of computational modelling in atrial fibrillation. Top panel illustrates clinical treatment strategies for managing atrial fibrillation. It includes catheter ablation, anti-arrhythmic drug therapy, and measures for stroke mitigation such as anti-coagulation. The middle panel demonstrates how computational modelling can inform AF treatment by showing that models can aid in understanding the arrhythmogenic substrate, planning model-based ablation strategies, and stratifying patients’ stroke risk. Lower panel presents the insights that can be derived from computational modelling in AF management, including improved understanding of AF mechanisms, optimized ablation targets, and better risk assessment for stroke.

Computational models provide a unique vantage point for understanding the pathophysiology of atrial arrhythmias and their impact on stroke risk.^68^ By integrating clinical data, imaging findings, and electrophysiological parameters, these models offer personalized insights into the mechanisms of arrhythmia and thrombus formation (*Figure 3*). Recent studies have shown the usefulness of *functionally similar* digital twins in predicting individualized stroke risk based on AF burden, atrial fibrosis patterns, and hemodynamic parameters.^69^ These models enable clinicians to tailor anti-coagulation therapy and rhythm control strategies to mitigate stroke risk while minimizing adverse effects. This type of computational work has also been applied in the context of probing relationships between structural remodelling and potential arrhythmia triggers or drivers,^70–72^ creating personalized treatment plans for catheter ablation of persistent AF,^15,73,74^ and predicting risk of arrhythmia recurrence following catheter ablation procedures.^75,76^ Nevertheless, there are still several key areas that require further exploration to advance our understanding and improve patient outcomes.

First, there is a need for continued refinement and validation of computational models to enhance predictive accuracy and clinical usefulness. This involves incorporating additional patient-specific data and refining algorithms to better capture the complexity of atrial arrhythmias and their impact on stroke risk. Additionally, further investigation into novel biomarkers and imaging techniques holds promise for improving risk stratification and identifying high-risk patient populations. From a clinical standpoint, prospective clinical studies are warranted to evaluate the effectiveness of personalized treatment strategies guided by computational models in reducing stroke risk in patients with atrial arrhythmias.

### Ventricular arrhythmias

VT treatment poses significant challenges, characterized by lengthy procedures, high patient risks, and unacceptable recurrence rates.^77^ Despite advancements in mapping and ablation technology, comprehensive characterization of the intricate scar substrate and associated electrical abnormalities that typically drive re-entrant VT remains incomplete. Integration of pre-procedural imaging to identify structural remodelling is being increasingly used to assist in the identification of slow conducting tissue isthmuses located within dense scar.^78^ LGE-MRI can robustly identify scar and surrounding border-zone tissue, enabling direct localization and quantification of intricate 3D channels (or ‘corridors’) through scar as ablation targets.^79,80^ On the other hand, CT defines chronic ischaemic scar based on regions of anatomical wall-thinning,^28^ However, this is not appropriate for the non-ischaemic population, and may not be robust in general given the latest evidence suggesting the importance of 3D scar architecture in VT circuits in these patients.^81^

**Figure 4:**
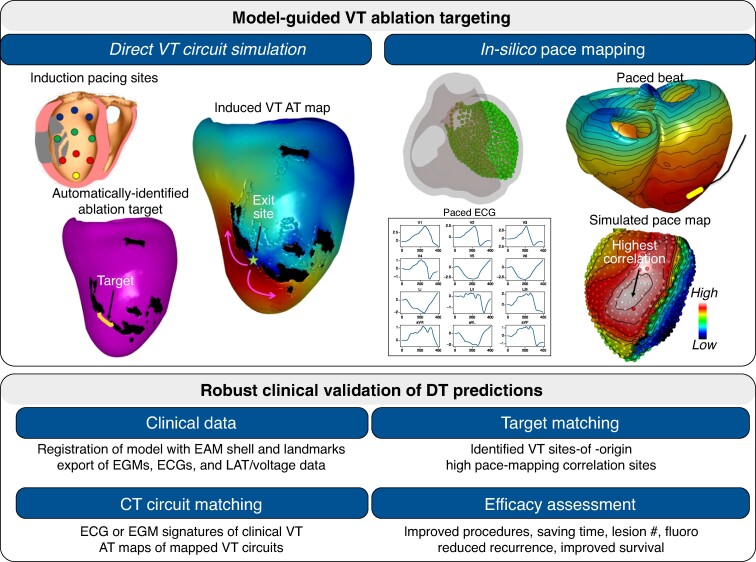
Clinical application of computational modelling for ventricular tachycardia. Top Panel illustrates model-guided VT ablation with the left section illustrating the process of direct VT circuit simulation, where induction pacing sites are used to identify the ablation target automatically. The simulation provides a visual map of the induced VT activation time, highlighting critical regions such as the exit site and target areas for ablation. The right section depicts *in silico* pace mapping, which involves the use of computational models to simulate pacing an identify regions of high correlation to pinpoint VT sites of origin, guiding precise ablation targeting.

Computational simulation has the unique advantage of probing functional implications of structural remodelling seen in imaging data to assess VT vulnerability and identify targets (*Figure 4*). Patient-specific image-based substrate models, using CT^18^ and LGE-MRI,^14,20,82–84^ can be used in conjunction with virtual VT-induction protocols, to uncover VT circuits and simulate corresponding ECG signatures. Building upon several initial works showing encouraging agreement with clinical data,^14,47^ careful and robust validation with clinical data of these modelling approaches must be performed. Such validation should come in the form of both quantitative comparison of ablation targets between CARTO maps (carefully registered with biventricular anatomical models) and in terms of finger-print ECG (or device EGM) signatures of the presenting clinical VT episode. Showing high correlations in this context would be key in the practical use of these models to accurately identify the clinical VT presented by the patient, gaining clinical confidence. Once validated, of course, these models then need to demonstrate their clinical utility, when integrated into a clinical workflow. Reductions in procedure time and complications, along with enhanced efficacy—measured by the absolute gold standard of reduced VT recurrence post-procedure—need to be demonstrated before these methods should be translated.

### Risk stratification

Identification of biomarkers to enhance stratification of risk for implantable cardioverter-defibrillator (ICD) implantation represents a significant clinical challenge, where it is widely acknowledged that the guideline-based compromised LV ejection fraction remains a highly non-specific metric of risk.

Image-derived biomarkers, primarily LGE presence on CMR, have been shown to be strongly associated with malign ventricular arrhythmias in both non-ischaemic^85,86^ and ischaemic cardiomyopathy cohorts.^87–89^ In this context, simulations enabled by digital twins may offer a valuable tool to uncover functional consequences of structural remodelling identified in the images. Applying virtual VT induction protocols to digital twin models offers the unique possibility to non-invasively assess complexity of scar substrate and its potential to sustain VT. Preliminary studies, all on relatively small ischaemic cardiomyopathy cohorts, have demonstrated the potential of virtual models to identify risk in post-ablation^83^ and ICD cohorts.^13,84,90^ Scaling these studies up to significantly larger cohorts at an *earlier* stage of their patient pathway (i.e. not ablation and ICD cohorts where elevated risk has already been identified), is critical to fully investigate the potential of digital twin approaches in making such life-and-death decisions. Simulations within such large cohorts required to provide robust evidence of their potential effective use represents a significant computational challenge.^90^ For the use of fully 3D bi-ventricular models, more rapid re-entrant path-finding approaches,^17^ as an alternative to computationally expensive mono-domain protocols may play an essential role.

## Envisioning the future

In our quest to create functionally equivalent mechanistic digital twin models that are continuously or periodically updated and offer reliable predictions of patient behaviour in real-world settings, research is still in its early phase. Advances towards translating the promising concept of digital twins into viable clinical applications are ongoing,^15^ but currently digital twin methodologies are mostly developed and used within the realm of academic research. There are significant challenges ahead to be addressed beyond pure research, involving also technological developments, regulatory work, and clinical studies. All of these carry significant economic costs requiring industrial engagement. While there are major initiatives promoting industrial engagement such as the Avicenna alliance (https://www.avicenna-alliance.com), and regulatory frameworks outlining a path forward,^91^ compelling success stories of economically viable and clinically beneficial applications are rare, and mostly limited to physiologically comparably simple modelling applications (https://www.heartflow.com/). While digital twins in industrial applications, e.g. as a medical device development tool is gaining traction,^5,6^ for leveraging digital twins in the clinic a further increase in industrial commitment is required. However, this will only be ignited once more credible evidence of clinical value is becoming available through clinical trials.

This section outlines a vision for the future of digital twins in cardiac electrophysiology, highlighting the necessary technological advancements, the integration of diverse data sources, ambitious future possibilities, and a research agenda to overcome clinical implementation barriers^92^ and achieve these goals.

### Key improvement areas

Digital twins in cardiac electrophysiology have the potential to revolutionize patient care by providing highly personalized and precise treatment plans. The goal is to develop functionally equivalent digital twins that can continuously integrate new data and provide real-time predictions and insights into a patient’s cardiac health. To realize this vision, several key areas require significant technological advancements:

1. Anatomical imaging: Enhancements in imaging modalities such as MRI and CT are crucial. This includes improved artefact suppression and reproducible medical image analysis to enhance anatomical fidelity. Robust differentiation of various structures and tissue types, correlated with their electrophysiological properties, is essential.
2. Automated pipelines: Developing fast, robust, and automated pipelines for creating accurate anatomical models is vital. These pipelines should minimize manual intervention and reduce operator bias.
3. Electrophysiology models: The creation of lightweight electrophysiology models that can execute in real-time or faster is necessary. These models should have reduced computational demands while maintaining high accuracy for model calibration and predict relevant clinical signals (ECGs and EGMs) to support a clinical like-for-like comparison.
4. Model calibration: Enhanced calibration techniques that build on global sensitivity analysis to identify the most influential parameters are needed. These techniques should aim for robust and unique parameter identification, possibly incorporating a priori constraints and uncertainty quantification to measure the reliability of predictions.
5. Data integration: A crucial aspect of future digital twins is seamless integration of data from various sources, including wearables. Events that might trigger a digital twin update include health interventions (ablation, drug change, and new comorbidity) and lifestyle changes (weight gain or loss, changes in smoking habits, and exercise routines). Continuous updates and parametrization are essential to maintain a digital twin’s accuracy and relevance. The integration of data from wearables, such as implanted cardiac devices, heart rate monitors, fitness trackers, and smartwatches, can provide real-time insights into a patient’s daily activities and physiological responses. This continuous flow of data can enable more precise and dynamic modelling.
6. Model validation: More rigorous regulatory-strength evidence is needed to demonstrate a match in the physiological response between patient and digital twin under conditions the model was not calibrated for. This is key to prove that the mechanistic nature of the models translate into correct and clinically useful predictions of patient responses to any treatment-related perturbations.

### Role of ML/AI in enhancing digital twins

This review primarily focuses on mechanistic models as the basis for digital twins. Nevertheless, we believe that complementary ML/artificial intelligence (AI) modelling methods are promising as a means of enhancing digital twins. We also anticipate ML/AI approaches will play an important independent role as purely data-driven tools for analysing exploding treasure troves of clinical and wearable data.^93–95^

ML/AI techniques may mitigate limitations of traditional biophysical models, wherein incompletely understood mechanisms are inherently not embedded in the model, but available data are abundant. By leveraging its ability to process large-scale and complex datasets, ML/AI can uncover patterns and relationships that may not be captured by mechanistic models alone. This extends beyond data analysis, offering robust methods to handle variability and uncertainty, which are critical in biological systems where patient heterogeneity remains a key challenge. Moreover, AI enables scalable, real-time updates of digital twins as new data become available, ensuring that models remain aligned with the evolving clinical profile of the patient. By integrating ML/AI into digital twin frameworks, the precision and adaptability of these models can be significantly improved, facilitating more accurate predictions and supporting personalized treatment strategies. For a review on the integration of mechanistic modelling with ML/AI techniques we refer to the manuscript from Alber *et al*.^96^

### Future goals and research agenda

Achieving true *functionally equivalent* digital twins represents a moonshot goal in cardiac electrophysiology. These models would continuously adapt to new data, predicting both acute response to therapy and long-term disease progression. Ambitious future possibilities include:

1. Predictive modelling: forecast the long-term evolution of cardiac diseases, helping clinicians to intervene proactively, at the right time, with the most effective treatment.
2. Personalized treatment: Tailored therapies to every unique physiological profile, optimizing outcomes, and minimizing adverse effects.
3. *In silico* trials: Conducting large-scale virtual clinical trials to test new therapies and medical devices, significantly reducing the need for extensive human trials.

To achieve these ambitious goals, the technological advancements listed above must be accompanied by advancements in real-time data collection. Sub-objectives in this space include those discussed above (e.g. related data gathering improvements in medical imaging, data interoperability, and clinical validation). Moreover, in the realm of wearable technology, it will be helpful to invest in the development of devices that can capture a wide range of physiological data with high accuracy and reliability. Lastly, it will be helpful to leverage artificial intelligence and ML to develop new algorithms for model calibration, data integration, and predictive analytics.

By focusing on these areas, digital twins can advance towards creating highly reliable and functionally equivalent models that will transform patient care, making precision medicine a reality.

## Conclusion

Digital twins represent a groundbreaking advancement in cardiac electrophysiology, offering unparalleled opportunities for personalized medicine and precision treatment. By creating dynamic, patient-specific models that integrate anatomical, electrophysiological, and clinical data, digital twins have enabled simulation and prediction of cardiac behaviour with unprecedented accuracy. Despite the challenges in achieving real-time updates and high-fidelity calibrations, the ongoing technological advancements in imaging, data integration, and computational modelling hold immense promise. The future of digital twins in healthcare envisions highly personalized treatment plans, predictive disease modelling, and large-scale *in silico* trials, significantly enhancing patient care and outcomes. Continued research and development in this field will be essential to fully realize the potential of digital twins, transforming them from a promising concept into a standard practice in cardiac electrophysiology.

## Data Availability

There are no new data associated with this article.
